# Chilling and Forcing From Cut Twigs—How to Simplify Phenological Experiments for Citizen Science

**DOI:** 10.3389/fpls.2020.561413

**Published:** 2020-09-04

**Authors:** Annette Menzel, Ye Yuan, Andreas Hamann, Ulrike Ohl, Michael Matiu

**Affiliations:** ^1^Ecoclimatology, Department of Ecology and Ecosystem Management, TUM School of Life Sciences, Technical University of Munich, Freising, Germany; ^2^Institute for Advanced Study, Technical University of Munich, Garching, Germany; ^3^Department of Renewable Resources, University of Alberta, Edmonton, AB, Canada; ^4^Geography Education, Institute of Geography, University of Augsburg, Augsburg, Germany; ^5^Institute for Earth Observation, EURAC Research, Bolzano, Italy

**Keywords:** climate change, phenology, bud burst, school project, science education, Shiny App

## Abstract

Low-cost phenological experiments with cut twigs are increasingly used to study bud development in response to spring warming and photoperiod. However, a broader variety of species needs to be tackled and in particular the influence of insufficient winter chilling deserves more attention. Therefore, we investigated if and how chilling requirements can be efficiently investigated by cut twigs and how this low-tech approach could be successfully implemented as a citizen science or school project. We conducted an experiment on bud burst and leaf development of *Corylus avellana* L. twigs, with natural chilling outdoors on a shrub (S) and another chilling treatment as cut twigs in containers (C), and subsequent forcing indoors. Subsampling of the number of cutting dates and number of twigs was used to infer minimum required sample sizes. Apart from insufficiently chilled twigs, ~80% of the twigs (both S and C) reached leaf out. For multiple definitions of chilling and forcing, a negative exponential relationship was revealed between chilling and amount of forcing needed to reach certain developmental stages. At least 5 out of 15 cutting dates or alternatively half of the 10 twig repetitions, but especially those mirroring low chilling conditions, were needed to describe the chilling-forcing relationship with some degree of robustness. In addition, for cutting dates with long chilling, i.e., from January onwards, freshly cut twigs (S) required significantly more forcing to reach bud burst than twigs from containers (C), although the effect was small. In general, chilling conditions of mature shrubs were well captured by cut twigs, therefore opening the possibility of chilling through refrigeration. We conclude that experimental protocols as outlined here are feasible for citizen scientists, school projects, and science education, and would have the potential to advance the research field if carried out on a large scale. We provide an easy-to-use Shiny simulation app to enable citizen scientists to build up a bud development model based on their own experimental data and then simulate future phenological development with winter and/or spring warming. This may encourage them to further study other aspects of climate change and the impacts of climate change.

## Introduction

Bud burst and new leaves in spring fascinate nature lovers on all continents. Their observations of spring phenology through citizen science networks are also widely used to track biological impacts of climate change around the world (Menzel, 2002; Beaubien and Hamann, 2011; Kobori et al., 2016). Yet, the environmental factors and plant physiological processes that control these spring development processes are still not fully understood. For temperate species, the interplay of cold winter temperatures that break (endo-)dormancy and warm spring temperatures forcing budburst is considered the main driver of the timing of budburst (Murray et al., 1989; Laube et al., 2014a; Polgar et al., 2014; Zohner et al., 2016). Additional cues associated with spring phenological events for some species include autumn temperature (Heide, 2003; Sparks et al., 2020), day length (Heide, 1993a; Heide, 1993b; Körner and Basler, 2010; Basler and Körner, 2012; Laube et al., 2014a), light quality (Brelsford and Robson, 2018), air humidity (Düring, 1979; Laube et al., 2014b; Zipf and Primack, 2017), or nutrient availability (Jochner et al., 2013a). Under natural *in situ* conditions it is difficult to disentangle these multiple and often co-varying environmental factors (e.g., Piao et al., 2019; Menzel et al., 2020).

In order to correctly predict budburst dates under climate change, the combined effects of cold winter temperatures (chilling requirements) and subsequent warm spring temperatures (heat sum requirements) must be considered (Chuine et al., 2016). This is an evolutionary adaptation to prevent budburst in autumn or mid-winter during unseasonal warm spells. But under climate warming, chilling requirements may not be satisfied in the future, thus preventing plants from timely budburst at the beginning of the growing season (e.g., Fu et al., 2015). Long-term *in situ* observations from phenology networks are, however, insufficient to disentangle the influence and relative importance of these two factors for different species and different geographic regions. Especially the underlying processes of chilling, i.e., the temperature response of buds during dormancy and dormancy break, are difficult to be derived from observations alone (Luedeling and Brown, 2011; Blümel and Chmielewski, 2012; Jochner et al., 2013b; Primack et al., 2015; Chuine et al., 2016).

One way to determine the effects of chilling and forcing is to experiment with woody plants under controlled conditions. Experiments with adult tree individuals under controlled (growth chamber) environments are technically challenging, however buds on twigs have been shown to serve as model units for tree individuals (Lebon et al., 2005; Basler and Körner, 2012; Laube et al., 2014a; Polgar et al., 2014; Primack et al., 2015), since excised twigs put in water may develop as they would on adult trees under normal conditions (Sønsteby and Heide, 2014; Vitasse and Basler, 2014). In contrast, tree seedlings or saplings are less desirable model units as they are known to differ in their budburst dates from adult trees (Augspurger, 2008; Vitasse et al., 2014). So far the influences of varying day length, humidity, forcing temperature and exposure time to natural chilling conditions, and freezing have been studied in factorial designed twig experiments (Laube et al., 2014a; Primack et al., 2015; Vitra et al., 2017). Mehlenbacher (1991) studied the chilling requirements of hazelnut inflorescences and vegetative buds by cutting shoots in the field at weekly intervals and forcing them in a warm greenhouse; rest completion was assumed when >50% of the buds started to develop after 3 to 4 weeks. However, horticultural studies have also exposed twigs to artificial chilling conditions for varying time periods after harvest (Gariglio et al., 2006; Jones et al., 2013; Sønsteby and Heide, 2014).

This low-tech, low-cost solution allows testing of many species. Consequently, phenological experiments on cut twigs have become more and more popular. So far, phenology experiments under controlled conditions have revealed that the influence of cold winter chilling and warm spring forcing temperatures is highly species-specific (Murray et al., 1989; Menzel et al., 2006; Laube et al., 2014a; Polgar et al., 2014; Zohner et al., 2016) and can also vary significantly among cultivars or genotypes within a species (Mehlenbacher, 1991; Gariglio et al., 2006; Sønsteby and Heide, 2014). Chilling requirements of trees may also differ among geographic regions (e.g., Mehlenbacher, 1991), leading to unexpected delays to phenology under climate warming, when chilling requirements are high (e.g., Heide, 2003; Ford et al., 2016). However, experimental scientific work was only able to capture a rather small sample of mainly northern temperate tree species, and broad inferences on regional or global phenological responses to climate change are not possible. A wider variety of species needs to be tested for solid generalizations, e.g., with respect to phylogeny or ecological strategies. Ideally, we should have experimental data of similar geographic and species coverage as we have observational data from citizen science networks for spring phenology.

Although chilling and forcing experiments are usually conducted by trained researchers in complex factorial experimental designs and are often aided by controlled environment chambers, the experiments are in principle straight-forward and could be conducted by citizen scientists at home who could thus easily help to fill some of these knowledge gaps by testing any woody species of personal preference (Primack et al., 2015). Data recording only needs a pen and paper, which is fast, easily standardized, and due to the beauty of the process highly self-rewarding. Installing and observing twig experiments during wintertime may be attractive for citizen scientists and pupils living in temperate regions, since these harbingers of spring bring attractive greenness into winterly homes. Moreover, cutting the St. Barbara twigs on December 4^th^ has been an old tradition dating back to medieval times (e.g., Gemminger, 1874; Michels, 2004). Nevertheless, the sustainable success of citizen science experiments may depend largely on their attractiveness, a clever set-up of the experiments and data management as well as on the participants’ motivation and long-term involvement.

For a successful large scale program of citizen science experiments, some research is required to develop standardized experimental protocols that can be carried out easily and yield robust results. Methods of applying chilling temperatures vary considerably, e.g., exposure of twigs to chill conditions under natural conditions and varying harvest dates, or application of chilling temperatures to cut twigs in freezers or refrigerators. It yet seems unclear which method is best suited to describe the chilling-forcing relationship properly. It remains especially unclear how often twigs need to be harvested during the dormant period, e.g., three (Laube et al., 2014a; Zohner et al., 2016), four (Polgar et al., 2014), or eight times (Murray et al., 1989) during winter, or as often as either weekly or bi-weekly (Chuine et al., 2016; Nanninga et al., 2017).

The objective of this study, therefore, was twofold. We first asked if the chilling requirements of woody species, in our case of *Corylus avellana* L., can be assessed *via* the excised twig method, using indoor and outdoor environments in lieu of growth chamber-based designs. Second, we investigated how sampling and experimental settings should be designed in order to describe the chilling-forcing relationship properly. We further tested if the twig method was a suitable tool to involve citizen scientists, reported minimum requirements, successes and drawbacks, and proposed a simulation tool to link experimental findings to potential climate change impacts.

## Material and Methods

### Experiment on Cut Twigs

**Figure 1:**
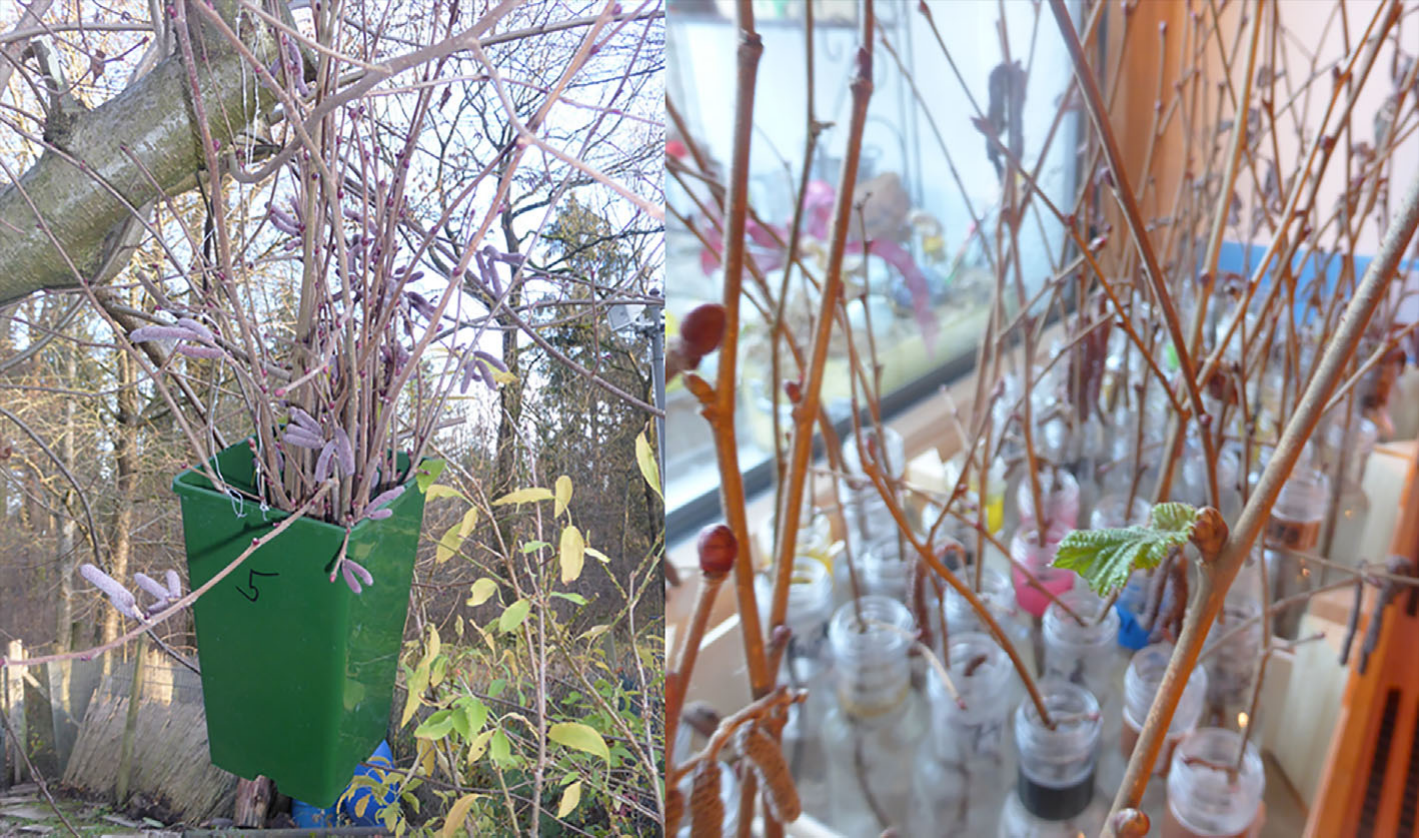
Container filled with twigs harvested on 21 November 2015 (left) attached to the *Corylus avellana* L. shrub outside (treatment “C”) as well as twigs in 0.1 L glass bottles filled with tap water on the windowsill inside (right).

The experiment was performed at the edge of a small town in southern Germany (~ 48.468° N 11.932° E), ~ 55 km northeast of Munich. In a garden adjacent to a forested area a 5 m tall hazel (*C. avellana* L.) shrub was selected. This hazel shrub had been regularly pruned in previous years; thus, uniform 2- to 3-year-old shoots branched at a height of 2 m and could be easily pruned again. The chilling-forcing experiment consisted of two chilling treatments outdoors, 1) natural chilling on the shrub (S) as well as 2) chilling of twigs that had been cut once on the first day of the experiment and then were stored outdoors in containers (C) fixed to the shrub (**Figure 1**). Treatment S represents the main experiment, while C was meant to explore the possibility of different experimental manipulations of chilling, e.g., in refrigerators, if cutting at the beginning of the trial does not have a major influence on twig development.

For both chilling treatments (S, C), spring forcing conditions were simulated by taking twigs indoors after different periods of chilling (**Figure 1**). More specifically, twigs were moved indoors to stop accumulation of chilling and to begin accumulation of heat sums (forcing) on 15 dates, beginning in 21 November 2015 until 14 March 2016 (see **Table 1**). As it would be anticipated for a citizen science study, the intervals were not always equal, which did not affect the analysis. Initial intervals were approximately 1 week until the end of January, subsequently intervals varied from 6 to 13 days.

**Table 1:** Summary of dates when natural outdoor chilling was terminated and twigs were brought to indoor forcing conditions.

Cutting	Date	Days since start	Difference in days to last cutting date
1	2015-11-21	0	
2	2015-11-28	7	7
3	2015-12-05	14	7
4	2015-12-12	21	7
5	2015-12-19	28	7
6	2015-12-27	36	8
7	2016-01-04	44	8
8	2016-01-10	50	6
9	2016-01-17	57	7
*	2016-01-20	60	
10	2016-01-25	65	8
11	2016-02-05	76	11
12	2016-02-14	85	9
13	2016-02-24	95	10
14	2016-03-08	108	13
15	2016-03-14	114	6
*	2016-03-22	122	

*denotes days when only phenological observations were made.

In order to implement the chilling treatment C, on 21 November 2015, the first day of the experiment, a few randomly selected branches were cut off, and 100 twigs of 50 cm length were harvested. These twigs were placed in three green 3-L plastic containers, perforated at the bottom to avoid waterlogging, but filled with moss to prevent rapid drying. All containers with the branch supplies for the subsequent forcing experiment were thereafter attached to the hazel with metal wires at a height of 2.5 m, so that they hung outdoors in positions comparable to those of the intact branches of treatment S acquiring natural chilling (**Figure 1**). On each of the cutting dates 2 to 15 (see **Table 1**), five twigs were taken out of this prepared storage, re-cut by ~15 cm at their base, cleaned, and placed in labeled 0.1 L glass bottles filled with tap water.

On cutting dates 1 to 15, ten 30 cm long twigs of the chilling treatment S were freshly cut from the hazel shrub, cleaned, and placed in labeled 0.1 L glass bottles filled with tap water. All bottles (S and C) were placed inside a residential house on the south-facing inner windowsill of the kitchen. Twigs were re-cut at the lower end on each cutting date and the water was exchanged regularly on every other cutting date.

### Phenological and Meteorological Data

Vitality assessment (i.e., twig status dead or alive) and phenological observations according to the Biologische Bundesanstalt Bundessortenamt und Chemische Industrie (BBCH) coding system (Meier, 2001) were done periodically throughout the experiment until 22 March 2016 (on all cutting and two additional dates, see **Table 1**). We observed the BBCH stages of bud development (principle growth stage 0) and leaf development (principle growth stage 1), i.e., 0 winter dormancy, 1 beginning of bud swelling, 3 end of bud swelling, 7 beginning of bud breaking, 9 bud shows green tips, 10 first true leaf emerged or mouse-ear stage, 11 first leaves unfolded, and 12 two first leaves. Mortality assessment was performed by carefully scratching the bark with a fingernail according to Seidel and Menzel (2016). The twigs were classified as dead when the cambium underneath was no longer green. This recording as well as the installation of the experiment (see *Experiment on Cut Twigs*) was primarily done by a 9-year-old schoolchild and her family under the supervision of the first author.

At the beginning of the experiment, two calibrated air temperature and humidity loggers (HOBO Pro v2, Onset Computer Corporation, Southern MA, USA) were placed both inside on the kitchen windowsill and outside fixed at a height of 2.5 m in the hazel shrub, where they measured these variables every 10 min (10 min temperature) in a shaded position.

### Statistical Analyses

Each twig of the experiment was assigned a unique ID, which referred to the chilling treatment of the twig (twig from shrub S, twig from container C), the week or date it was taken inside to start forcing according to the predefined cutting scheme (cutting date of S or date of transfer of C, respectively) and a consecutive number. A few missing BBCH stages in the course of the observations and one missing HOBO logger day were imputed linearly. We determined for each cutting date the final BBCH stage of a twig, which was either reached when it was identified as dead, when it was removed from the experiment with leaf out (BBCH>=12), or at the end of the observations (22 March 2016). Chilling and forcing accumulation began 21 November 2015, the start of the experiment.

Chilling was determined in three ways: sum of chilling days based on the 10 min temperature recordings (temperature < 5°C, fractional days possible), sum of days with daily mean temperature < 5°C, and number of days outside. Forcing was equally determined in three ways: degree days based on the 10 min temperature recordings (temperature ≥ 5°C, fractional degree days possible), degree days based on daily mean temperature ≥ 5°C, and number of days inside. We also tested a threshold of 0°C and obtained similar results, thus only the results with a 5°C threshold were presented here. Chilling and forcing amounts were calculated from the start of the experiment until the date on which the twig was classified as dead, or having reached BBCH stage of 7, 9, 11, or leaf out.

The relationship between chilling and forcing was assumed to be exponential, as in Murray et al. (1989):

forcing=a+b*exp(c * chilling)+є

where a, b, and c are estimated parameters, and є are errors assumed to be normally distributed. The associated fits were plotted along the chilling *vs.* forcing plots.

In order to compare forcing requirements of the two chilling treatments, fresh cut twigs (treatment S) and container stored twigs (C), we calculated an ANOVA-style model with forcing depending on chilling, treatment (S or C), and their interaction. Chilling was inserted as a factorial (dummy) variable to be able to test differences for each chilling value, which corresponds to the week of cuttings. Since error variances were not identical for all chilling values, we used a GLS (generalized least squares) model, in which parameters were added to model different error variance (one for each chilling value). *Post-hoc* tests were done separately for chilling and treatment, by holding the other variable constant. P-values were adjusted for multiple testing. Some data were discarded when the number of twigs for a cutting date (chilling days) was too small (e.g., BBCH 9 or 11 and less than 25 chilling days).

In order to develop an optimal experimental protocol for implementation by citizen scientists, we studied the effect of reducing the number of observations. These sensitivity tests were performed by subsampling from the experiment and simulating the respective chilling-forcing relationships. First, we studied the effect of less frequent cutting dates. From the initial 15 cutting dates, we selected 8, 7, 5, and 4 dates, because they could be evenly distributed with uniform sampling periods (i.e., every 2, 3, or 4 weeks). For each reduced cutting numbers, we also adjusted the starting week (for 7 cuttings, starting week 1 and 2; for 5 and 4 cuttings, starting week 1, 2, and 3). The effect of the less frequent observations was compared visually using the exponential fits.

Second, we evaluated the effects of fewer branches within treatment S. At each cutting date, 10 twigs were cut from the shrubs, and we tested the effect of having only 2 to 9 twigs instead. For each number of fewer twigs (2-9), we took random samples of the 10 twigs at each cutting date, and fit the exponential curve to this subsample. This procedure was repeated 1,000 times, and the different exponential curves were plotted along the original fit to the full data set for comparison.

All computations were performed using R statistical software, version 3.6.2 (R Core Team, 2020), additionally using packages data.table (Dowle and Srinivasan, 2019), emmeans (Lenth, 2020), ggplot2 (Wickham, 2016), and the tidyverse collection (Wickham et al., 2019).

## Results

### Meteorological Conditions

**Figure 2:**
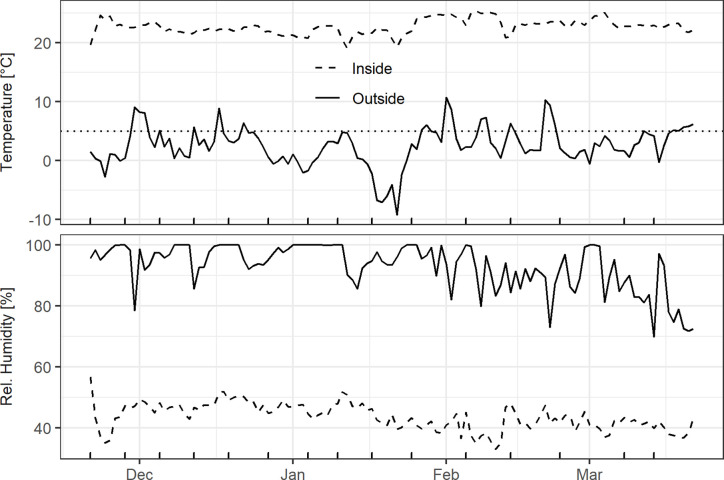
Daily mean air temperature and air humidity outside at a height of 2.5 m in the hazel shrub as well as inside on the south-facing windowsill. Dotted line in the top panel represents the 5°C threshold used for chilling/forcing and the x-axis ticks at the bottom indicate the cutting dates.

The inside HOBO logger indicated that temperature and humidity conditions were fairly stable, with the daily mean temperature fluctuating around 22.5°C and relative humidity ranging between 40 and 50% (**Figure 2**). The outside weather conditions were characterized by alternating chilling and forcing conditions. The daily mean air temperatures in December, February, and March were mostly above 0°C outdoors, whereas a longer cold spell occurred in mid-January. Relative humidity was mostly above 90% until the end of January, and gradually decreased from February onwards (**Figure 2**).

### Twig Development and Survival

**Figure 3:**
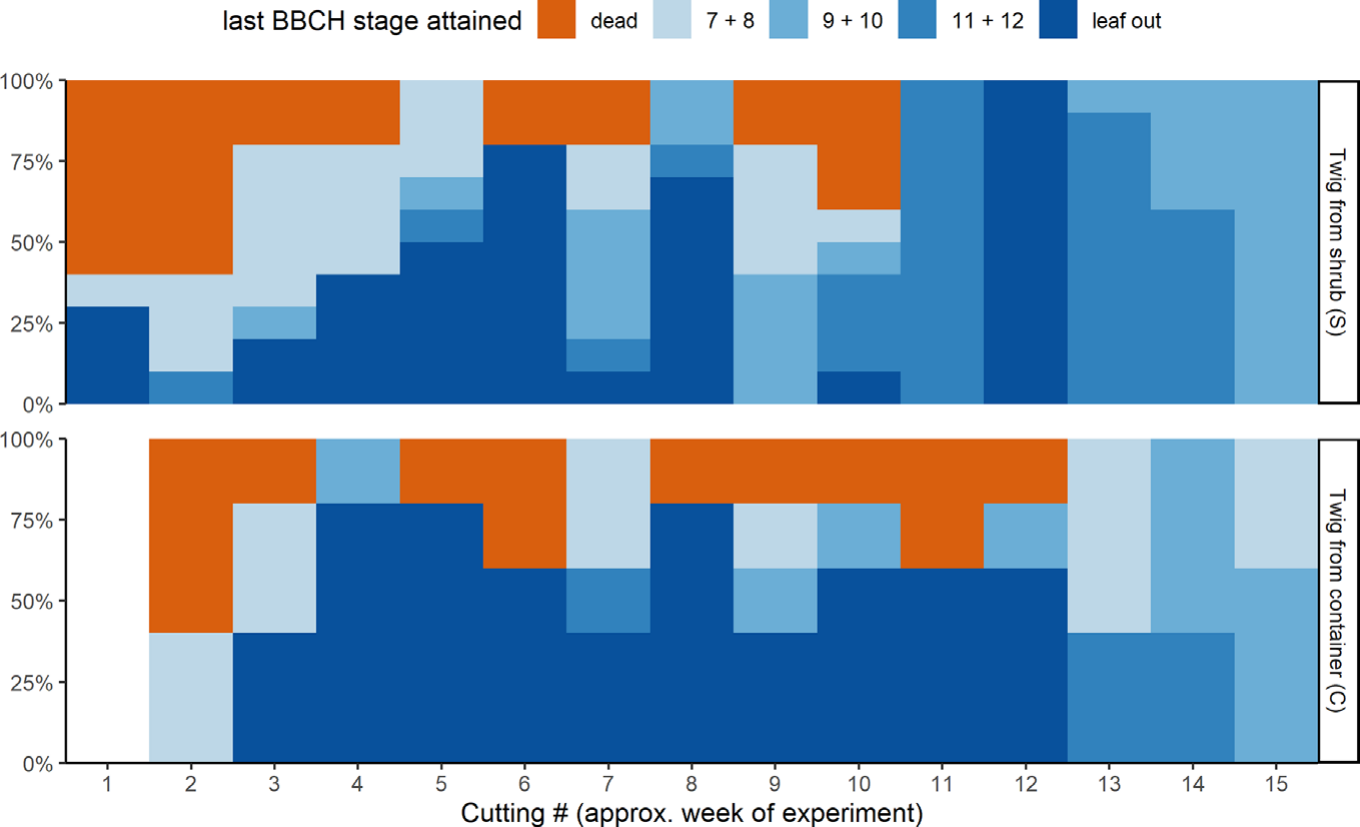
Last developmental stages attained of the ten freshly cut twigs from the shrub (S, top) and of the five twigs taken from the container storage (C, bottom) for the different cuttings.

Not all twigs reached the last BBCH developmental stage recorded (**Figure 3**). The percentage of twigs that fully developed beyond BBCH 12 depended on the cutting date (S) or the date on which the twigs were brought inside from the containers (C), respectively. About 40% of the twigs from cutting dates 1 and 2 (S) and cutting date 2 (C) in November with almost no chilling failed to develop and were later classified as dead. For all other cutting dates, less than 20% of the twigs did not develop by the end of the experiment. Of the twigs brought inside from week 3 to 12, a higher percentage of those previously stored in the containers reached full leaf out. Twigs that were cut at one of the last cutting dates in March (13 to 15) usually had already been close to BBCH 11, whereas twigs from the containers were somewhat delayed in their development outdoors. The development of those twigs could be observed till stage BBCH 12 and beyond, since no twigs had to be removed any more as fully leafed twigs due to space restrictions.

### Analysis and Simplification of Chilling-Forcing Relationships

**Figure 4:**
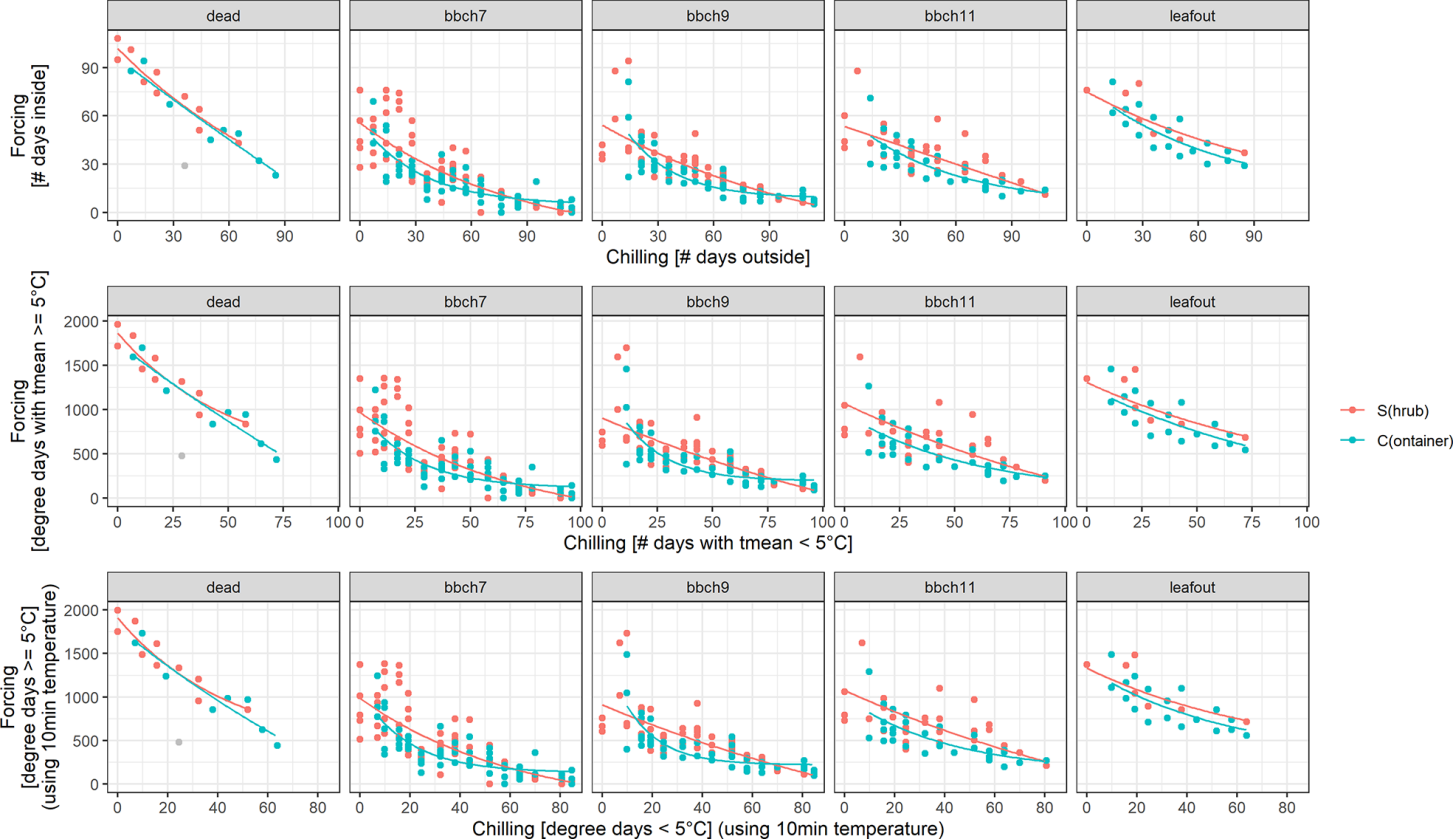
Chilling-forcing relationships for the three different chilling and forcing definitions as well as for the two twig treatments (S freshly cut from the shrub, C previously stored cut in containers outside). Lines are the fitted exponential functions (see *Material and Methods* for equation). The gray point in the panel “dead” was considered an outlier and was excluded from the fitting.

For all treatments (S, C), BBCH stages and definitions of chilling and forcing, an inverse relationship was clearly shown between acquired chilling and amount of forcing required to reach certain development stages (**Figure 4**). For all stages (dead, BBCH 7, 9, 11, leaf out) this reciprocal relationship was almost identical regardless of the definition of chilling and forcing used (5 or 0°C threshold, based on 10 min or daily temperature values, as the number of days or daily sums). Even the simplest approach, defining the days outside as chilling days and the days inside as forcing days, led to similar relationships.

The later the developmental stage (BBCH 7, 9, 11), the higher the forcing accumulated up to that point in time for a given cutting date/chilling. Considerably higher forcing sums for leaf out and dead twigs can, however, be distorted by the fact that the final decision to remove a twig as fully developed or dead obviously took some time. For the later cutting dates (longer chilling), the apparent chilling-forcing relationship was fairly linear, whereas earlier cutting dates with less chilling led to the typical negative exponential function.

**Figure 5:**
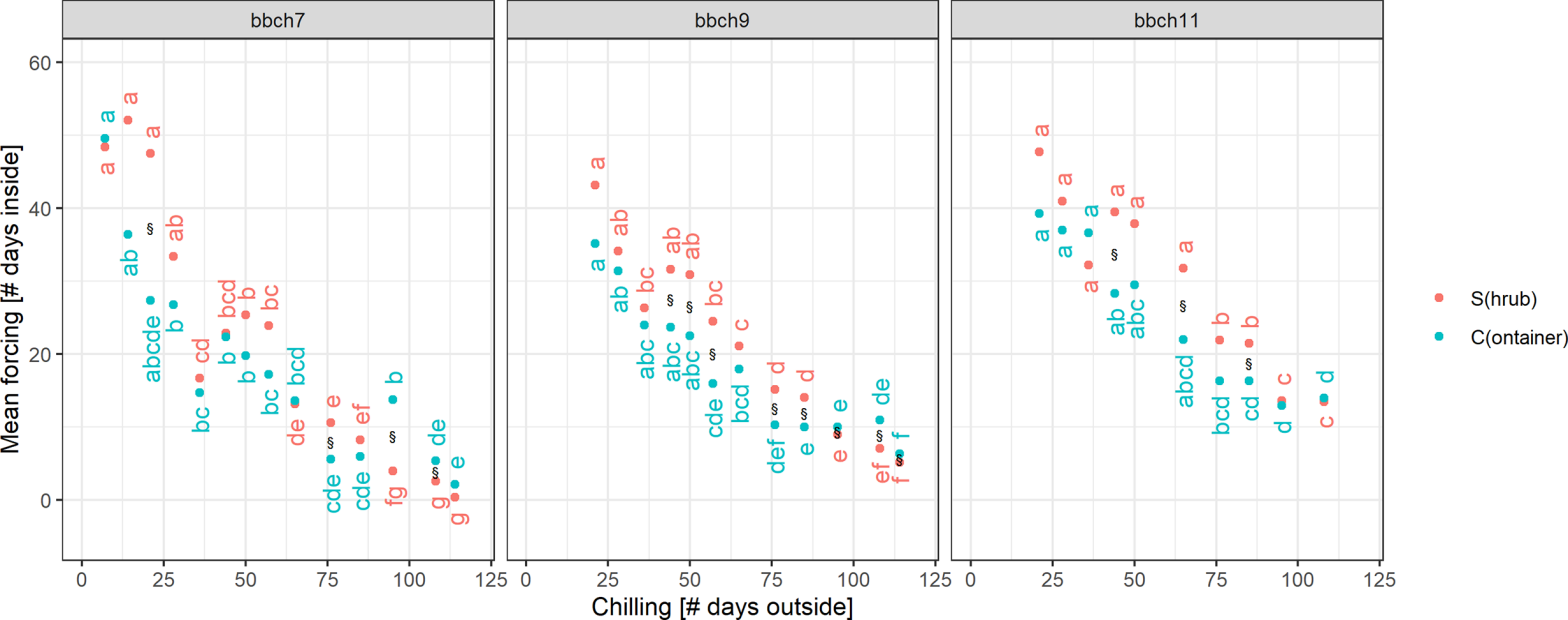
Significance of differences in chilling and forcing at three developmental stages. Dots indicated the mean forcing (days inside) and mean chilling (days outside), § indicates a significant difference in forcing between twigs freshly cut from the shrub (S) and taken from the containers (C) at the single cutting dates. Small letters indicate significant differences of forcing between the cutting dates (or chilling days outside), separately for twigs from shrub (S) or containers (C). Mean estimates and tests based on an ANOVA-style model (see *Material and Methods* for more details), significance based on p-value < 0.05, and p-values adjusted for multiple testing.

For median chilling/cutting dates, the required forcing for twigs previously stored in containers (C) was slightly less than for freshly cut twigs (S). However, formal testing for significant differences revealed that not all of these differences were statistically significant (**Figure 5**). In case of BBCH 7, cutting dates with long chilling in particular led to significant differences. All twigs that were cut on day 44 or later of the experiment, i.e., from January onwards, required significantly more forcing for twigs freshly cut (S) to reach BBCH 9 than for C. The same result was observed for BBCH 11, although fewer cutting dates exhibited these significant differences. Within the treatments (S, C), the forcing sums of up to about five consecutive cutting dates were not statistically significant from each other.

**Figure 6:**
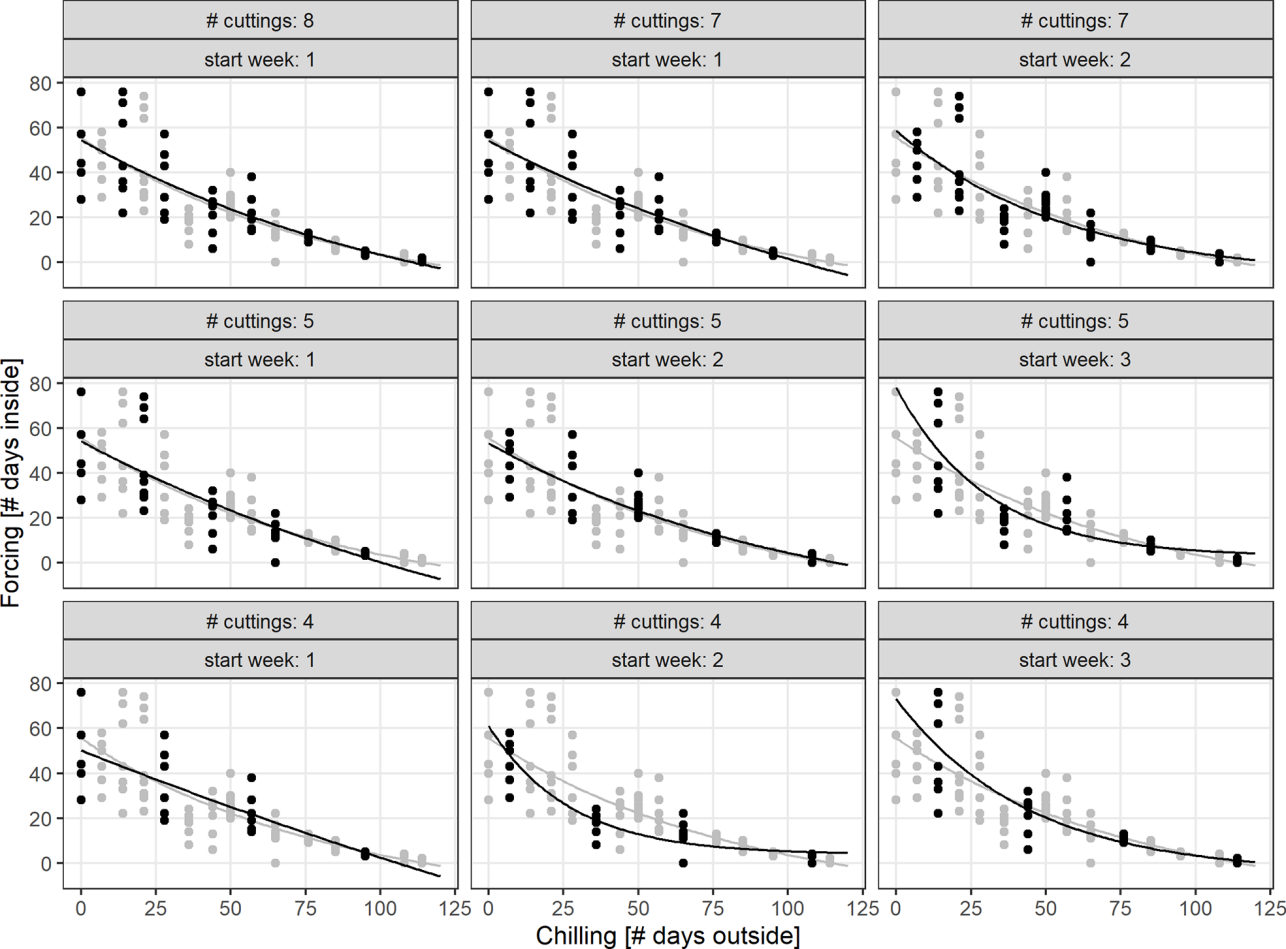
Simulation of Biologische Bundesanstalt Bundessortenamt und Chemische Industrie (BBCH) 7 chilling-forcing relationships for less intensive monitoring (less cuttings/observations and different starting dates; indicated in panel titles). The full dataset (gray) is for twigs from shrub (S), the black dots are the simulated smaller datasets.

Simulations of the chilling-forcing relationships based on days outside and inside suggested that at least 5 to 8 cutting dates may be needed to derive stable relationships comparable to the results of the full set of 15 cutting dates from late November to early March. It was particularly important that initial sampling dates representing low levels of chilling support a solid representation and should therefore not be missed or discarded. The fewer cutting dates there were, the greater the influence of the first recordings in November on the overall chilling-forcing relationship was (**Figure 6**).

**Figure 7:**
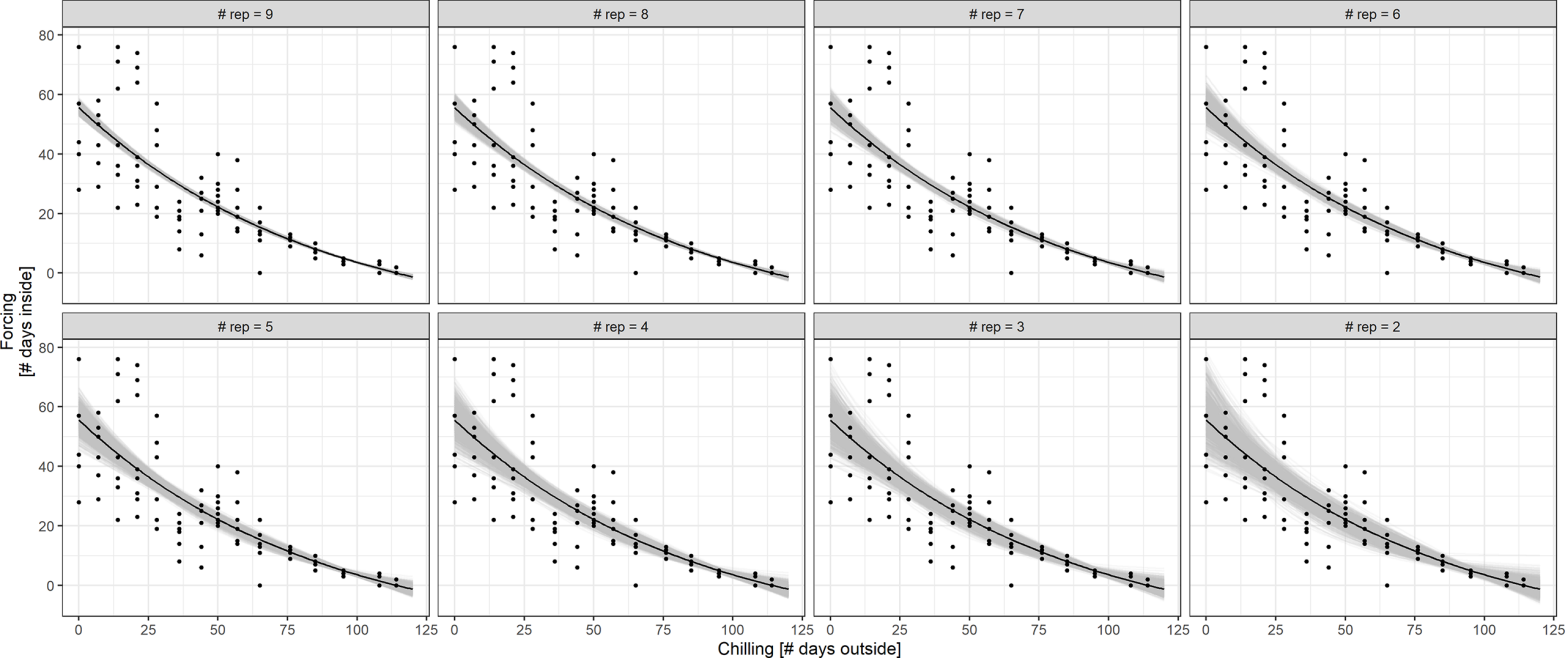
Simulation of BBCH 7 chilling-forcing relationships for less repetitions (fewer twigs cut) in treatment S. The full dataset with the associated exponential fit is shown in black (10 twigs/points per chilling value; overplotting since multiple points with same values). The gray lines indicate fits to random subsamples with 9 to 2 repetitions instead of 10 (1,000 random subsamples per repetition value).

An alternative way to reduce the workload for the citizen scientists would be to reduce the number of twigs that are harvested and observed in the house. **Figure 7** displays the results of BBCH 7 simulations based on treatment S with 10 twigs at each cutting date. The exponential fit of the chilling-forcing relationship is quite stable for 9 to 7 repetitions. For 6 and 5 repetitions, the fits deteriorate on the side with little chilling which is particularly important for estimating impacts of future climate change with reduced chilling in connection with winter warming. With less than 5 repetitions, there is a considerable variation in fits.

## Discussion

### Practicability for Citizen Science/School Projects

This study demonstrated that a phenology experiment could be successfully conducted in the home of a citizen scientist. Several points addressed in the paper may facilitate the implementation of twig experiments. Twig experiments do not require complicated and expensive equipment, but only a pair of pruning shears, if applicable plastic containers for outdoors, glass bottles, permanent markers, paper, and pencil. Manipulation of chilling and forcing temperatures can easily be achieved by staggered cutting dates and different indoor conditions (heated room, unheated room, and refrigerator). In our study the indoor air temperature fluctuated only slightly, and these fairly constant indoor conditions can be assumed for houses with central heating. In our study the outside chilling conditions were also (by chance) quite stable during winter. Consequently, the chilling-forcing relationship was described equally well by the number of days inside (in lieu of calculating forcing degree days) *versus* number of days outside (in lieu of using exact chilling degree days). However, in other years, strongly fluctuating outside conditions may be possible, so that recording of temperatures can be useful and is therefore recommended. We were able to measure this reference data, but it has to be assumed that in large scale citizen science networks this won’t be always the case. Then, gridded daily temperature records could be used, and indoor temperatures could be assumed constant, based on an average of several readings. Alternatively, nearby weather station data or interpolated data products could be used, which are usually freely available from national or regional weather services. Although these may not account for local microenvironments, they still represent the major weather situation and would stabilize the growing-chilling relationship, thus allowing accurate inferences on the magnitude of chilling requirements for a particular species and region.

Unfortunately some issues may also impede a successful implementation. Studying different species or treatments takes up quite a bit of space, which may not be available in many households. In our case, 15 times putting 15 twigs (10 from the shrub and 5 from the containers) in the house resulted in more than 140 twigs, which had to be observed simultaneously in late January and early February. In our trial space restrictions led to twigs beyond BBCH 12 being removed and classified as full leaf out. Other interested citizen scientists could not participate due to lack of space in their apartments. Therefore, carrying out these projects in schools with more spacious classrooms and a larger floor space may be an alternative. However, schools being closed during the obligatory winter holidays (Christmas, New Year’s Eve) would result in an unintentional interruption of observations and supervision of the experiments. Furthermore, a reduction of the heating during these school holidays can lead to an inhomogeneity in the forcing conditions applied. Citizen science projects could be implemented in other locations (i.e., non-school or home settings), too, such as senior or community centers.

The choice of appropriate species may depend on the availability of specimen in the vicinity. Species with earlier budburst allow welcome activities during the dark and dormant winter season. Unfortunately some of these species produce allergenic pollen (e.g., the genera *Corylus, Alnus*, and *Betula*), but their release could be avoided by removing male catkins. In case of severe allergic predispositions, these species should be avoided.

### Suitability in Science Education

The twig experiment conducted in our study was not accompanied by formal educational surveys. Nevertheless, we assume that these experiments are well suited for science education in the context of climate change, as they deal with a relevant question in a project-driven, vivid, and interactive way. A twig experiment can be easily integrated into everyday life and literally can be carried out “in the own backyard” or home in this case. These hands-on activities motivate participants, and promote the acquisition of knowledge and scientific core competencies. The approach described is thus a textbook example of “inquiry-based learning (IBL)” which enables participants to learn about climate change impacts in their immediate surroundings (Brumann et al., 2019). In our study, observations of the participating student (motivation, work input) and a later interview supported this positive assessment. Especially the “hands-on” installation of the experiments and the cutting of twigs were welcome physical activities.

The interplay of hands-on and minds-on activities and the degree of self-activity can be varied in order to increase and harness the potential of such citizen scientists’ experiments. Participants could formulate and test their own hypotheses, e.g., on environmental drivers of bud burst and leaf development. They could formulate their own ideas, try out new experimental settings or incorporate other species in future trials. Consequently, depending on their specific prerequisites or needs during their learning process, participants can complete a typical scientific research cycle in whole or in part, e.g., by developing research questions and hypotheses, selecting and applying appropriate methods, and analyzing, interpreting, and presenting the results (Huber, 2009; Reitinger, 2013; Pedaste et al., 2015). In this way, they will acquire considerable competence in scientific methods and experiments.

In school projects, the teachers provide the pupils with appropriate assistance. These adequate measures of guidance are important for the success of inquiry-based learning processes (see Lazonder and Harmsen, 2016). In general, all citizen scientists must be supported in documenting their observations and in their analyses. Therefore, an easily understandable observation manual with many illustrations as well as data entry tables (printed or computer-based) for noting down observations should be provided. In the medium term, an internet platform on which the observations can be systematically uploaded and evaluated can also support participants in their analyses. However, it is not required that all participants carry out statistical analyses. The above-mentioned inverse relationship between the acquired chilling and the amount of forcing required to reach certain development stages can be shown without formal analyses as the results can be understood by looking at simple graphs of the results. Especially younger students can also work with the simplest approach described above and define the days outside as chilling days and the days inside as forcing days.

Our study design required a high level of motivation over a considerably long period of time for school children. Thus, the question of how frequently and when twigs have to be harvested or how many twigs are needed per cutting date is of central importance. Savings at this point would reduce not only the workload, but also the space required (see *Practicability for Citizen Science/School Projects*). We have shown that the cutting dates (chilling treatments) can be reduced from weekly to bi-weekly, but especially the early cutting dates were necessary to describe the chilling-forcing relationship as accurately as possible. Similarly, halving the number of repetitions/twigs would still yield robust relationships as long as low chilling data are sufficiently represented. However, both ways of reducing the workload imply at the same time that the experiment would still last almost 4 months and that additional motivational incentives might be needed to ensure that participants carefully follow the regular phenological observations and twig care.

Finally, measures to adapt the twig experiment to the specific prerequisites and needs of the participants suggest positive motivational effects in line with the self-determination theory (SDT) addressing the factors autonomy, competence, and relatedness (Van den Broeck et al., 2010; Thomas and Müller, 2014; Dettweiler et al., 2015). Following a given experimental setup and a fixed observation scheme may not suggest a high degree of autonomy, but reworking the experimental setup and formulating new hypotheses to be tested allow for autonomous decisions. Relatedness can be strengthened if the experiment is conducted together with family, friends or in a school context. The experience of competence can result from the sense of achievement of having made one’s own scientific discoveries. However, positive effects are not only to be expected in terms of *perception* of competence, but also in terms of actual development of competences, such as scientific knowledge in plant phenology, dendrology, and micrometeorology, as well as expertise in methodological skills in the sense of the scientific (propaedeutic) method (conducting experiments, collecting and analyzing data, presenting results).

This exemplary experience of the nature of science (McComas, 1998) can in turn contribute to a positive development of the participants’ epistemological beliefs, i.e., their individual views on the genesis, ontology, meaning, justification and validity of knowledge in science (Priemer, 2006). This is particularly beneficial in the context of climate change, where “fake news” is not uncommon and more understanding of science and scientific work is needed.

### Chilling Experiments With Twigs

For different definitions of chilling and forcing as well as for the two treatments (S, C) the well-known negative exponential relationship as proposed e.g., by Murray et al. (1989) and many others afterwards, could be confirmed; however, in our described prototype citizen science experiment, only for one donor plant and only for 1 year. Therefore, all technical results have to be assessed with caution. The related research question was whether chilling requirements of plant species can be appropriately studied by cut and experimentally manipulated twigs. Previous studies showed that twigs can be used as a proxy for adult trees’ phenological behavior, i.e., budburst timing (Vitasse and Basler, 2014). Vitasse and Basler (2014) compared the thermal time, i.e., the accumulation of degree days above 5°C since Mar 1^st^, to reach budburst for cut twigs put in water bottles directly positioned underneath the corresponding trees. In their study, the bud and leaf development of donor trees (adults and saplings) and their corresponding cuttings were well in parallel, with a slighter (mostly non-significant) delay of cuttings. However, using cut twigs needs to be evaluated as an experimental technique not only for studying forcing conditions, but also for investigating chilling requirements.

Vegetative buds of 6 out of 10 twigs cut on November 21^st^ and 28^th^ did not develop and were later classified as dead. This mortality rate seems high, however according to Mehlenbacher (1991) vegetative buds of hazel cultivars need 365–480 to 1,395–1,550 h of accumulated chilling (between 0 and 7°C) for >50% of the buds to develop. His conclusion was that the chilling requirement of vegetative buds is a major consideration in determining the area of cultivar adaptation. Therefore it is most likely that the chilling accumulated until the end of November in our experiment was not sufficient to complete rest. Equally for later cutting dates, some twigs did not develop, but more investigations are needed to verify whether this is related to the individual donor tree or is a general feature to be possibly overcome by more frequent recuts at the twig base or additives to the water.

We demonstrated that the chilling requirement of buds of a mature shrub was well captured by cut twigs stored outside in containers as the chilling-forcing relationships were similar between the two treatments. This finding allows proceeding with different experimental manipulations of chilling, e.g., in refrigerators. Interestingly, for identical chilling temperatures outside and forcing temperatures inside, twigs previously stored in the containers (C) needed less forcing days inside than the freshly cut twigs (S). The effect has to be related to the cutting as such, e.g., cut and interruption of transport, e.g., of dormancy promoting signals mediating the temperature response (Singh et al., 2016) or of water, if enhanced drying supported acquired chilling as Laube et al. (2014b) suggested. These differences were statistically significant, but small in magnitude for several, but not all of the cutting dates. Thus, more scientific chilling experiments are needed to get further insight into these slight differences in chilling of cut twigs *versus* intact branches. In addition, before making a broad recommendation for the use of cut twigs in citizen science experiments, trials should be conducted with more plant species.

## Outlook

We find that phenology experimentation with cut twigs could be a useful tool for education and outreach as Primack et al. (2015) proposed. Such trials also have potential to advance the field through large scale citizen science networks and secondary school projects by studying chilling (and forcing) requirements. These activities could contribute not only to education in phenology, dendrology, and weather, but also help to raise awareness of climate change and its impacts on nature.

**Figure 8:**
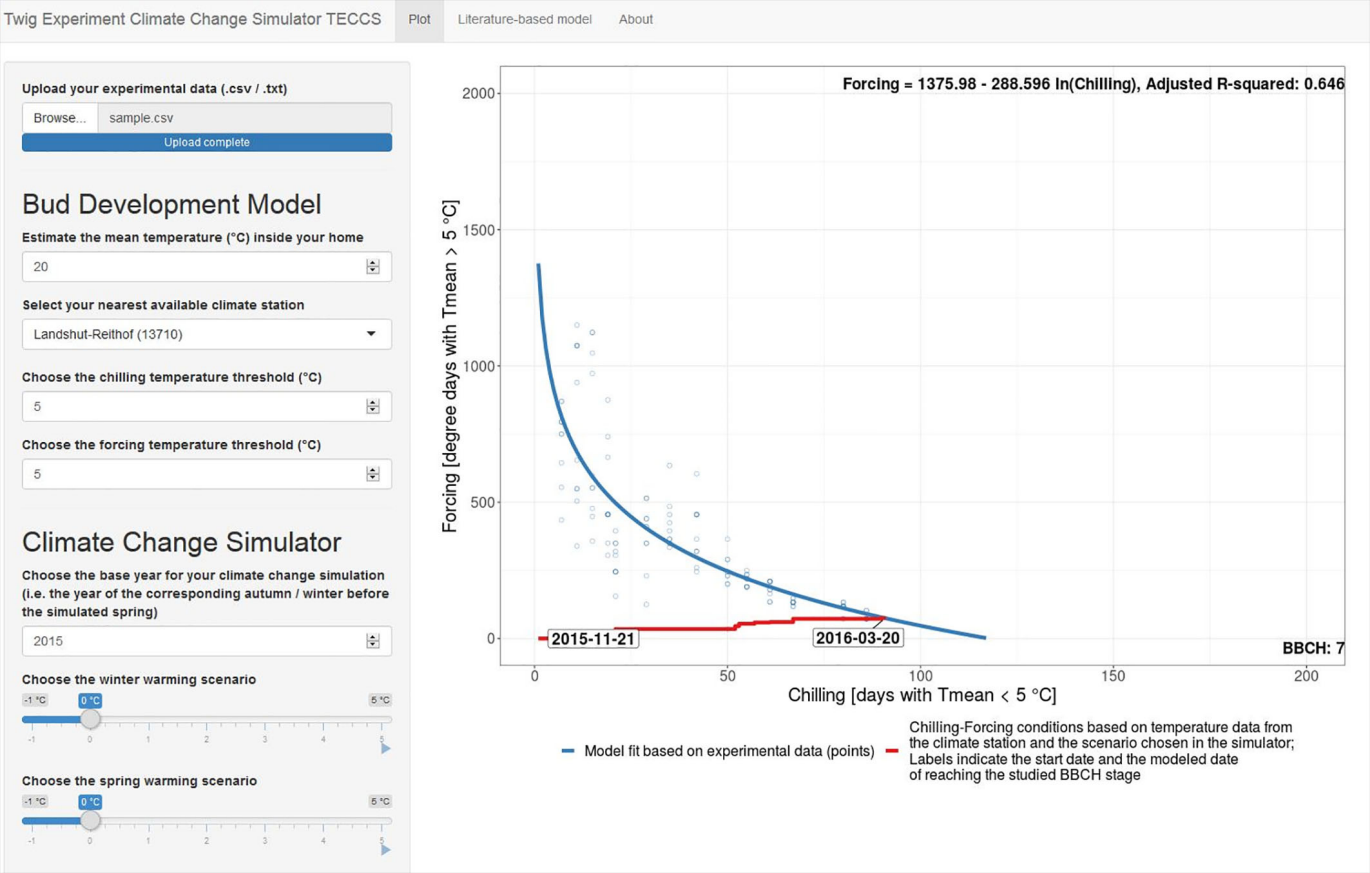
Shiny App Interface of the twig experiment climate change simulator (TECCS). The Shiny App is available at www.baysics.de as well as https://github.com/yuan-oekoklima/TECCS.git for the source code. Instructions on the twig experiment for TECCS are provided in **Supplementary Material**.

For this, we developed the easy-to-use simulation tool TECCS (twig experiment climate change simulator) to study potential effects of winter and/or spring warming on budburst dates (**Figure 8**). This Shiny App allows citizen scientists 1) to upload her/his twig observation data in predefined formats (.txt/.csv, corresponding templates provided in **Supplementary Material**), 2) to easily import corresponding outdoor temperature data from the nearest climate station and to estimate forcing conditions by indicating the mean indoor room temperature, 3) to fit a chilling (CD) forcing (GDD) model on the uploaded data based on the equation GDDcrit = a +b *ln CD, 4) to vary CD and GDD thresholds from the default 5°C threshold, and 5) to simulate future bud development for a chosen base year and winter and spring warming scenarios between −1 and +5°C. Once the temperature-based chilling-forcing relationships have been estimated on their own experimental data, the TECCS simulation tool thus allows the citizen scientists to autonomously analyze in terms of model building and simulations to answer important questions, such as “what is the likely effect of a 1°C winter warming and a 2°C spring warming on the timing of bud development?”. The TECCS Shiny App (version 1 for Bavarian climate station data) is available at www.baysics.de as well as the source code available at https://github.com/yuan-oekoklima/TECCS.git.

## Data Availability Statement

The raw data supporting the conclusions of this article will be made available by the authors, without undue reservation.

## Author Contributions

AM designed the study, and assisted the installation and running of the experiment conducted by our citizen scientist family. MM performed the statistical analyses. YY contributed the Shiny app, and UO the didactical part. AM wrote the first draft of the manuscript, which all authors critically checked and improved by distinct contributions. All authors contributed to the article and approved the submitted version.

## Funding

This data was analyzed in the framework of the project BAYSICS (Bavarian Citizen Science Portal for Climate Research and Science Communication) which is sponsored by the Bavarian State Ministry of Science and the Arts in the context of the Bavarian Climate Research Network (bayklif).

## Conflict of Interest

The authors declare that the research was conducted in the absence of any commercial or financial relationships that could be construed as a potential conflict of interest.
