# Supplementary material for: Chilling and Forcing From Cut Twigs—How to Simplify Phenological Experiments for Citizen Science
Source: Front Plant Sci. 2020 Sep 4;11:561413. doi: 10.3389/fpls.2020.561413 (PMC7498619; doi:10.3389/fpls.2020.561413)
Supplement: Supplementary file 1 [file DataSheet_1.pdf]

Instructions on the twig experiment for TECCS

Background

This twig experiment allows citizen scientists to study the influence of climate change on bud burst dates. The amount of warming (= forcing) that buds of woody species need to develop in spring is determined in relation to cold winter temperatures (= chilling) that are needed to break winter rest. This species-specific relationship is important to know since warming under climate change may further advance bud burst dates due to the faster achieved forcing or could even delay bud break when chilling in warmer winters is not sufficient.

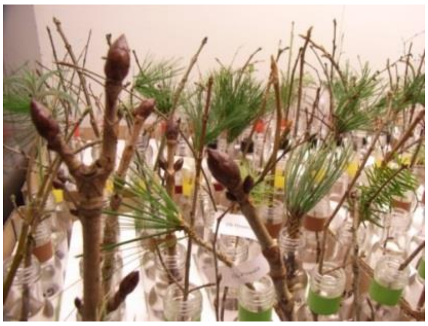

Preparation

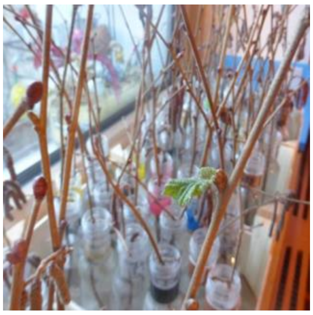
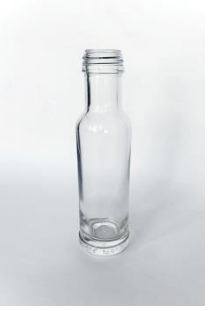
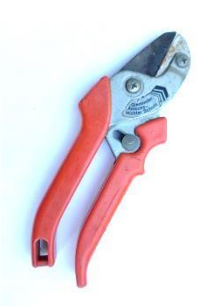
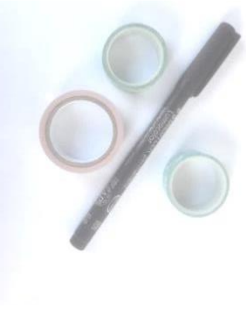
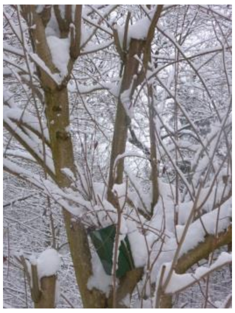

You need

1. A not too dark windowsill in a continuously heated room in your home
2. Glass bottles or containers to store the twigs
3. Sharp garden shears to cut the twigs
4. Adhesive tape and waterproof pen to label branches or bottles
5. A nearby tree or shrub to cut at least 50 twigs during winter

Experiment

Timeline

Start your experiment on any day in late November or early December when trees or shrubs have lost their leaves. This is "**Day 0**". Choose a day of the week on which you have some free time.
Cut off new additional twigs every week, preferably on the same day of the week, and observe all twigs which are inside on your windowsill. These dates are then **Day 7, 14, etc.** of the experiment.
Later, from January onwards, it is sufficient to cut new twigs only every second week; however instead it would be wise to observe bud development twice a week.

Cutting and maintenance of twigs

Cut 5 to 10 twigs of 30 cm to 50 cm length with a sharp shears, bring them inside your home and put them into the glass bottles filled with tap water. Label twigs (if you have several in one container) or the glass bottle (if there is only one twig) with an ID consisting of the Day of the experiment (14) and a consecutive number (e.g. B-14-03).

Each week, exchange the water in the bottles and recut from time to time at the base of the twig. If twigs have fully unfolded their leaves to the final size, they can be removed.

Observations of bud development

On each date of the timeline (Day 0, 7, 14, …) observe the bud development according to the defined BBCH stages. Note down for each twig the most advanced stage reached.

Example European Beech	BBCH Code Description
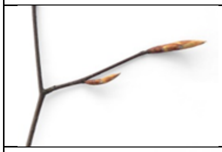	BBCH 0<br>Closed buds in winter rest
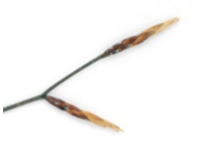	BBCH 1<br>Beginning of bud swelling
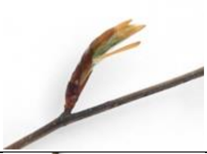	BBCH 7<br>Beginning of bud breaking
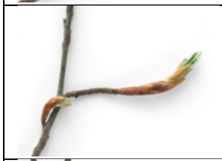	BBCH 9<br>Green leaf tips 5 mm above bud scales
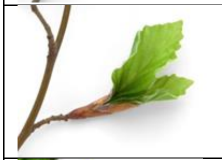	BBCH 11<br>First leaves unfolded, leaf bottom not yet visible
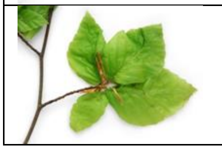	BBCH 19<br>First leaves fully expanded

The observation list then should look like this:

Twig ID B-14-03

Day of the experiment	BBCH	
14	0	
21	0	
28	1	
35	1	
42	7	
45	7	
49	9	and so on….

In order to produce the input table for the TECCS Shiny App, choose the BBCH stage you want to analyze, e.g. BBCH 7.

Then, for each twig extract the earliest date BBCH 7 was reached, e.g. Day 42 for Twig Day14 #3.

This twig would then have 14 days outside (since it was cut on Day14 of the experiment) and 28 days (42-14) inside.

The TECCS input of this twig would at the end look like this:
"Start of experiment: 2019-11-20"
"BBCH: 7"
"Days_outside","Days_inside","Branch_id"
14,28,"B-14-03"
